# Adult Hemophagocytic Lymphohistiocytosis in Sub-Saharan Area: A Retrospective Study of 26 cases

**DOI:** 10.7759/cureus.7258

**Published:** 2020-03-13

**Authors:** Ngoné Diaba Diack, Baidy Sy Kane, Seynabou Fall, Abibatou Sall, Abdoul Karim Daher, Moustapha Niasse, Nafy Ndiaye, Boundia Djiba, Fatou Samba Ndiaye, Abdoulaye Leye, Abdoulaye Pouye

**Affiliations:** 1 Internal Medicine and Endocrinology, Pikine Teaching Hospital, Dakar, SEN; 2 Internal Medicine, Aristide Le Dantec Teaching Hospital, Dakar, SEN; 3 Hematology, Dalal Jamm Teaching Hospital, Dakar, SEN; 4 Biology Laboratory, Dalal Jamm Teaching Hospital, Dakar, SEN; 5 Nephrology, Aristide Le Dantec Teaching Hospital, Dakar, SEN; 6 Rheumatology, Aristide Le Dantec Teaching Hospital, Dakar, SEN

**Keywords:** hemophagocytic lymphohistiocytosis, adult, tropical environment

## Abstract

Introduction and background

Hemophagocytic lymphohistiocytosis (HLH) is a condition caused by inappropriate stimulation of macrophage cells with hemophagocytosis. This paper aims to describe its diagnostic specifics and etiology and seeks to identify the factors that affect its prognosis in the black African adult population.

Methods

A retrospective multicentre study was carried out in three medical units in Senegal: Department of Internal Medicine at Pikine Teaching Hospital, and Department of Internal Medicine and Department of Nephrology at Aristide Le Dantec Teaching Hospital; the study covered the period from January 1, 2012 to March 30, 2015. This study included patients aged 18 years and older with a Hemophagocytosis Score (HScore) of ≥202 (with probabilities of acquired HLH of >90%). The data was obtained through medical records.

Results

In total, 26 patient files were included. The average age of the patients was 41 years, with a male-to-female ratio of 2.25:1. Fever and cytopenia were frequent. Other clinical signs included peripheral lymphadenopathy (69.2%), hepatomegaly (53.8%), splenomegaly (34.6%), neurological disorders (34.5%), and respiratory disorders (15.3%). Thrombocytosis was noted in three cases. Renal involvement was present in eight patients, with one case of collapsing glomerulopathy. The bone marrow aspirate revealed myelodysplasia in 12 patients. The dominant etiologies of HLH were hematological malignancies and infections. The mortality rate of HLH was 73%. Male gender and non-etiological targeted therapy were significantly associated with mortality. However, the age of <40 years in patients and current systemic disease in some cases were correlated with survival. The use of etoposide had no significant impact on the prognosis of our patients.

Conclusion

A high rate of male predominance, important central nervous system disorders, myelodysplasia, and paradoxical thrombocytosis were found to be the distinct features of adult HLH in our study population.

## Introduction

Hemophagocytic lymphohistiocytosis (HLH) involves the inappropriate stimulation of macrophage cells with hemophagocytosis [1]. Generally, it occurs in children with primary immunodeficiency. In adults, HLH often appears as a result of infectious disease, autoimmune disease, or malignancy. HLH is an increasingly recognized disorder in adults [2]. The diagnosis of acquired HLH in adults is often difficult with a broad differential diagnosis. Furthermore, therapeutic decision-making continues to be on the basis of clinical experience and expert opinion [2].

In Sub-Saharan Africa, there have been very few studies on HLH in adult patients [3-5]. The observations from these studies show some clinical and biological atypia that could indicate the specificity of HLH for black African people. Therefore, this study focuses on clinical, biological, and cytological presentations, etiological profile, and outcome factors related to HLH in the black African adult population.

## Materials and methods

A retrospective multicentre study was carried out in three medical units in Senegal: Department of Internal Medicine at Pikine Teaching Hospital, and Department of Internal Medicine and Department of Nephrology at Aristide Le Dantec Teaching Hospital; the study covered the period from January 1, 2012 to March 30, 2015. The data was obtained through medical records.

**Figure 1:**
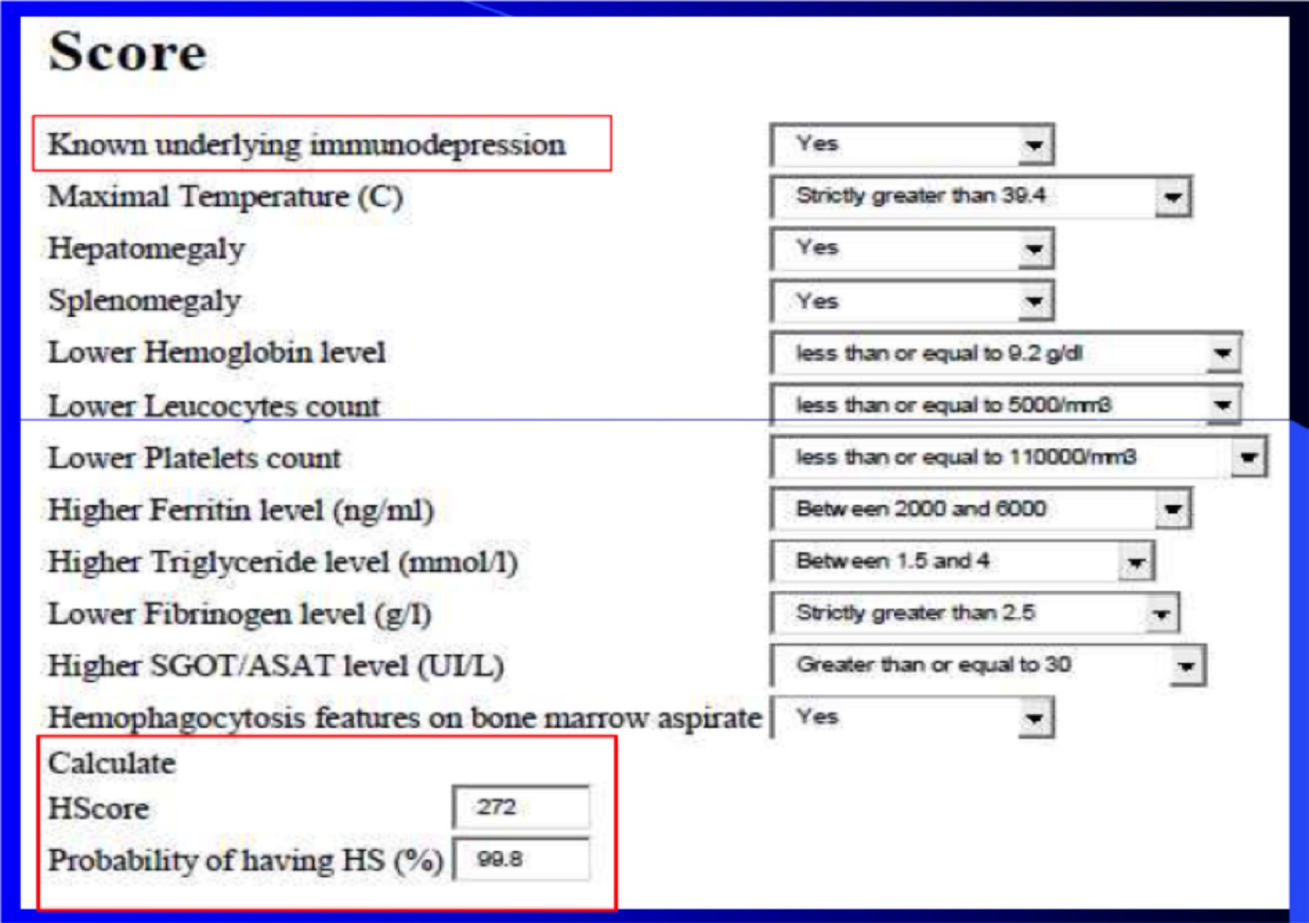
Example of the calculation probabilities of reactive HLH with Hemophagocytosis Score HLH: hemophagocytic lymphohistiocytosis; SGOT: serum glutamic-oxaloacetic transaminase; HScore: Hemophagocytosis Score; HS: hemophagocytic syndrome

This study included all patients aged 18 and older who were hospitalized during the study period with a Hemophagocytosis Score (HScore) of ≥202 (with probabilities of acquired HLH of >90%) (Figure 1) [6]. Patients with lost or incomplete medical records (n = 3) were not included in this study.

For each included patient, the epidemiological, clinical, biological, cytohistological, therapeutic, and evolutionary data were noted in a record chart. Criteria for the diagnosis of systemic diseases were based on Systemic Lupus International Collaborating Clinics criteria, American College of Rheumatology/European League Against Rheumatism 2013 classification criteria, and American-European 2002 Consensus Group criteria for lupus, systemic scleroderma, and Primary Sjogren's syndrome, respectively [7-9]. We used IBM SPSS Statistics for Windows, Version 20.0 (IBM Corp., Armonk, NY) for statistical analyses. Continuous variables were presented as means and standard deviation. Categoric variables were presented as proportions. For the analytical study, cases classified as “died” were compared with those classified as “survival” (all patients who were alive until the date of the last evaluation in April 2016 were included) using the Fisher exact test for categoric variables. A p-value of <0.05 was considered statistically significant.

## Results

Series description

Overall, 26 medical records were collected (18 men and eight women), with a male-to-female sex ratio of 2.25:1. The mean age of patients was 41 ±20.2 years old. Patients younger than 40 years old represented 61.5% of the study population.

Clinical and biological data

The different clinical signs recorded in patients included in our series are represented in Table 1. All patients experienced fever, which was hectic in approximately 75% of cases. Nine cases of central neurological signs were noted with a normal cytochemical and bacteriological exploration of the cerebrospinal fluid (CSF). Cerebral MRI performed on one of those patients was normal. Regarding the blood count, cytopenia was constant. Anemia was noted in all patients with an average hemoglobin level of 6.1 g/dL. Autoimmune hemolytic anemia was noted in six patients. Thrombopenia was present in 17 patients and was associated with a hemorrhagic syndrome in five patients. Thrombocytosis was recorded in three patients. The other biological abnormalities recorded in our patients are mentioned in Table 2.

**Table 1:** Main clinical signs of HLH recorded in our series HLH: hemophagocytic lymphohistiocytosis

Clinical signs	N (%)
Fever	26 (100)
Adenopathies	18 (69.2)
Hepatomegaly	14 (53.8)
Splenomegaly	9 (34.6)
Serositis	10 (38.4)
Neurological signs (conscious disorders, convulsions)	9 (34.5)
Pulmonary signs (dyspnea, cough, respiratory insufficiency)	4 (15.3)

**Table 2:** Main biological signs of HLH recorded in our series HLH: hemophagocytic lymphohistiocytosis; CRP: c-reactive protein; SR: sedimentation rate at the first hour; LDH: lactic acid dehydrogenase; N: number of patients in whom biological investigations were performed; n: number of patients in whom biological abnormalities were noted

Biological manifestations	N (%)	n/N (%)
High CRP	26 (100)	25 (96.2)
Elevated SR	21 (80.7)	19 (90.4)
Hyperferritinemia	23 (88.4)	23 (100)
Hypofibrinemia	19 (73)	3 (15.7)
Hemostasis disorders	22 (84.6)	7 (31.8)
Hepatic cytolysis	23 (88.4)	13 (56.5)
Hepatic cholestasis	15 (57.6)	13 (86.3)
Hypertriglyceridemia	20 (77)	16 (80)
Hyponatremia	22 (84.6)	18 (81.8)
Renal function disorders	26 (100)	8 (30.7)
High LDH	21 (80.7)	17 (90)

**Figure 2:**
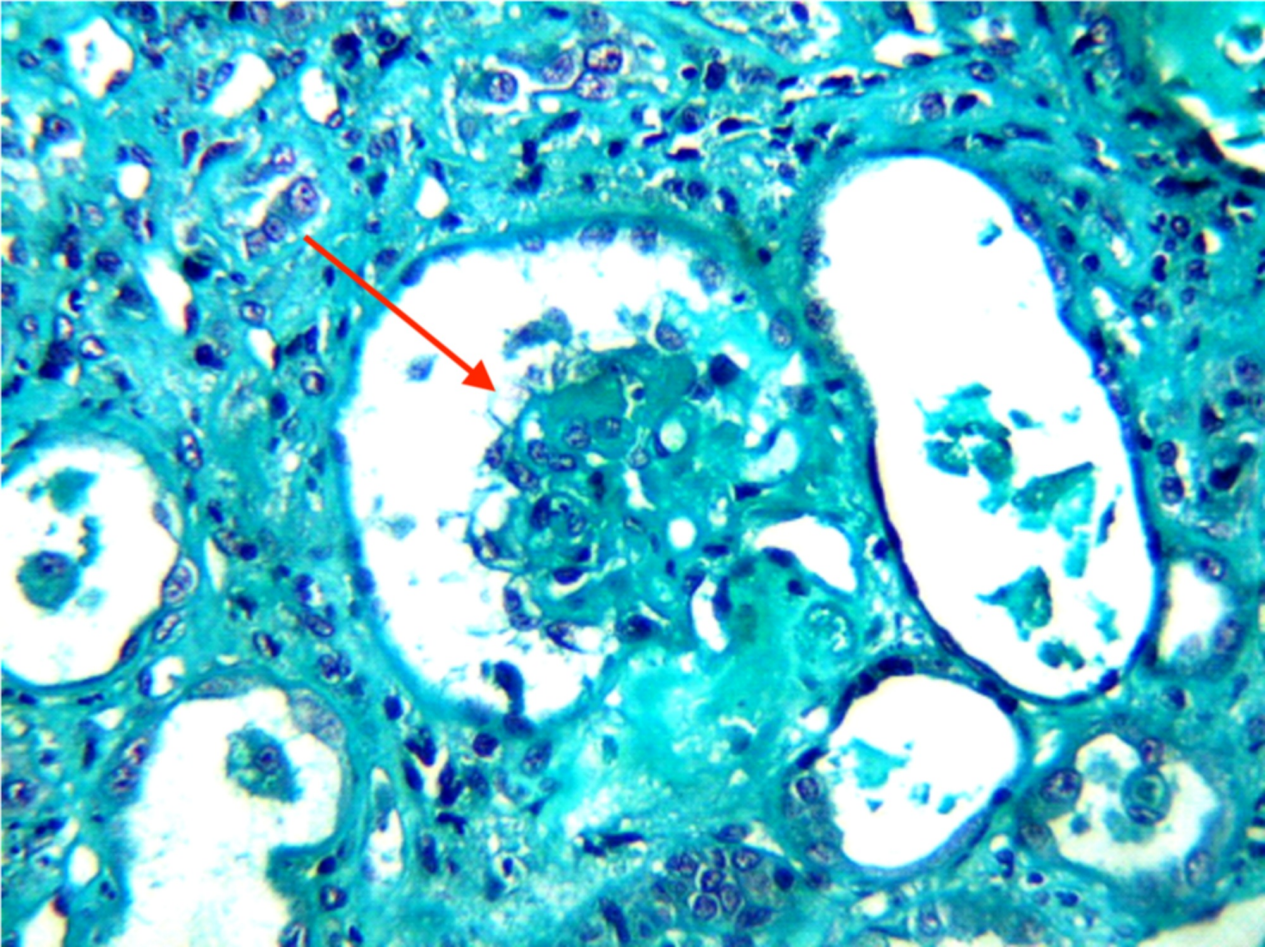
Collapsing focal segmental glomerulosclerosis Renal histology (Masson's trichrome; gross x 200) of a patient that shows a glomerulus (red arrow) in the center of the microphotography characterized by a global collapse of the flocculus surrounded by vacuolized dysmorphic podocytes

The increases in triglyceride levels, lactic acid dehydrogenase levels, and hepatic enzymes were moderate. Renal impairment markers (e.g., proteinuria, hematuria, aseptic leukocyturia) were recorded in all cases. Acute renal failure was noted in eight patients. Glomerular nephropathy was found and featured an impure nephrotic syndrome or rapidly progressive glomerulonephritis (RPGN) in four patients in our study. The renal histology of one of these patients found a collapsing focal segmental glomerulosclerosis (Figure 2).

Cytological and etiological data

**Figure 3:**
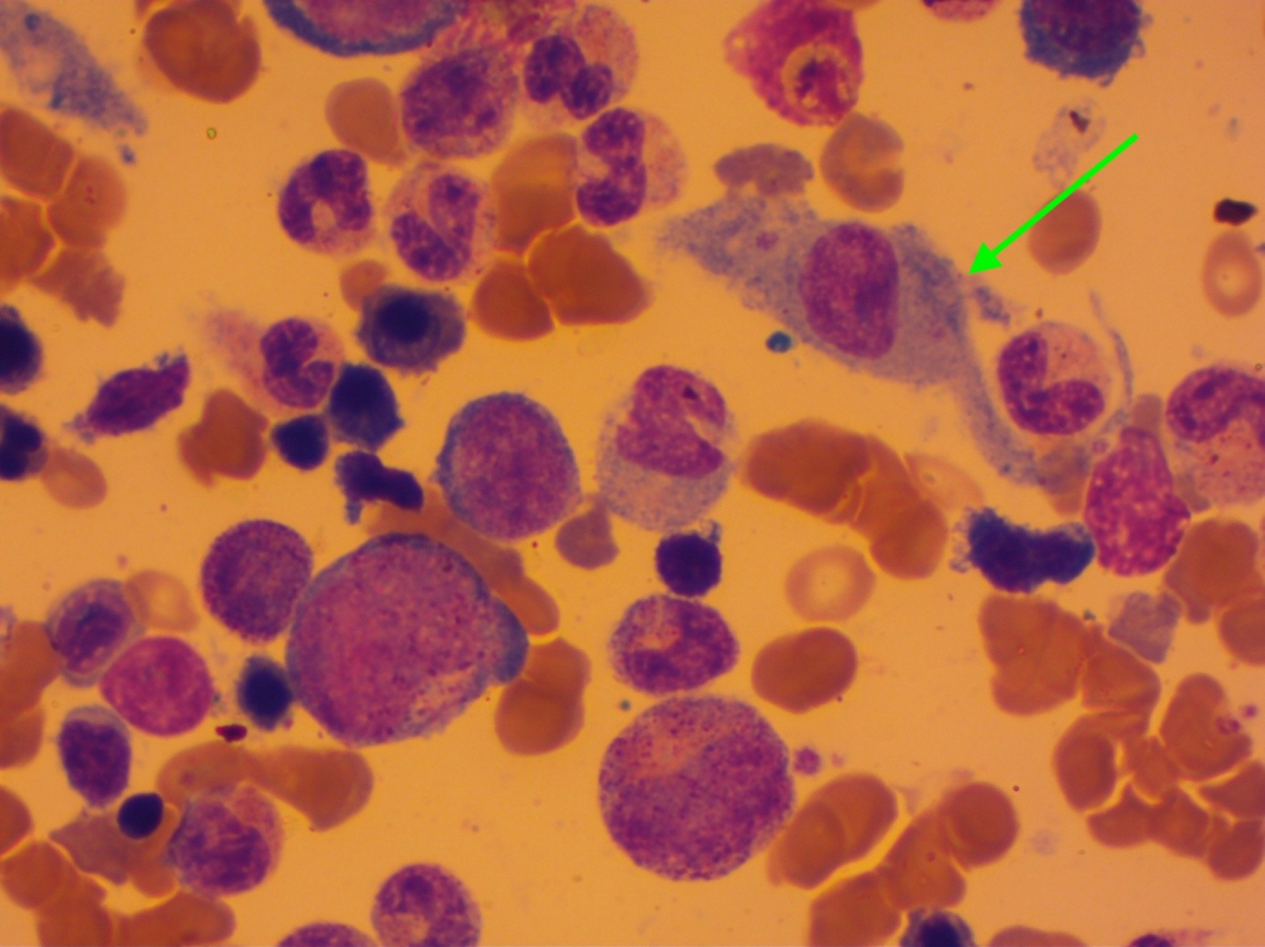
Macrophage activation Cytology of the medullar fluid showing an activated macrophage (green arrow) engulfing a hematopoietic cell (erythrocyte)

**Figure 4:**
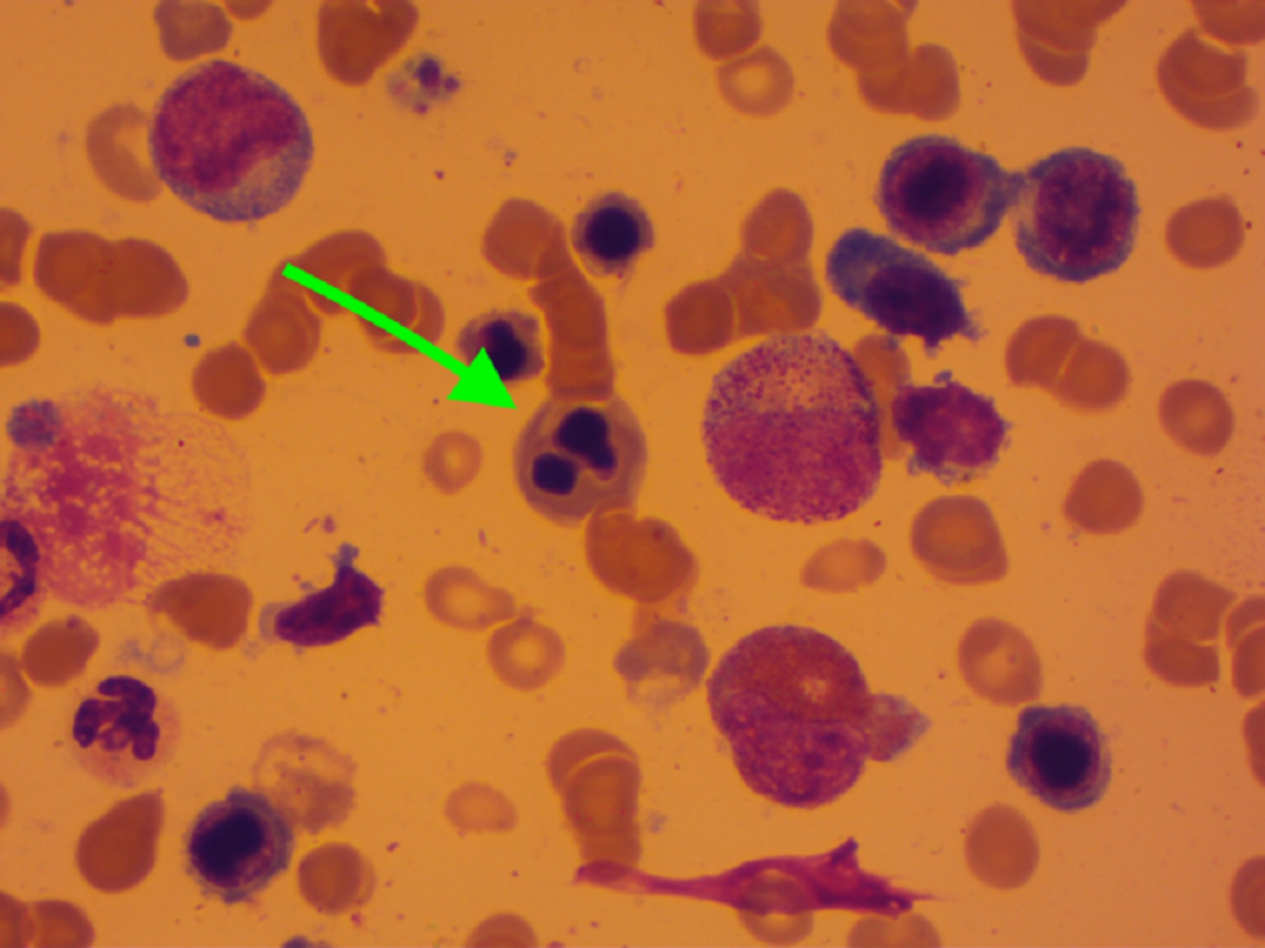
Dysmyelopoiesis Medullar cytology of a patient showing a dysmyelopoiesis type of karyorrhexis (green arrow)

The myelogram revealed a hemophagocytosis in 76% of cases (Figure 3) and showed dysmyelopoiesis in 12 patients. The dysmyelopoiesis essentially involved the erythrocyte line with abnormalities such as karyorrhexis (Figure 4), nucleo-cytoplasmic maturation asynchronism, dyskaryosis, binucleated erythroblasts, and basophilic punctuations. The presence of degranulated cells and giant cells was noted. A medullar plasmacytosis was also found in eight patients, outside of a myeloma context.

For the field study, the HLH triggering factor and lymph node biopsy were helpful in eight cases, and the myelogram in two cases. The conditions and/or factors of the patients are noted in Table 3. In two patients, the cause of HLH was not identified.

**Table 3:** Main fields and/or triggering factors of adult HLH found in our series HLH: hemophagocytic lymphohistiocytosis

Etiological framework	Main etiologies
Hematologic malignancies (11 patients, 42.3%)	Hodgkin's disease (four cases)
	Diffuse large cell B lymphoma (three cases)
	T-cell lymphoma
	Burkitt’s lymphoma
	Acute myeloid leukemia
	Multiple myeloma
Infections (12 patients, 46.1%)	Bacteria: tuberculosis (three cases), atypical mycobacteriosis, pyomyositis, sepsis by Enterobacter and Burkholderia cepacia
	Virus: Epstein-Barr virus, hepatitis B, human immunodeficiency virus
	Parasite: malaria
	Mycosis: systemic aspergillosis
Systemic diseases (six patients, 23%)	Lupus (two cases)
	Scleroderma
	Primitive Sjogren's
	Microscopic polyangiitis
	Multicentric Castleman disease
Primary immunodeficiency (one patient, 3.8%)	Common variable immunodeficiency
Unfound etiology (two patients, 7.6%)	

Therapeutic and evolutionary data

The specific treatment of HLH is found in corticotherapy (bolus of methylprednisolone for three days, followed by oral prednisone). Etoposide (VP-16) was administrated to seven patients. It was associated with corticotherapy in four cases. Specific treatment of the causal pathology (chemotherapy, anti-infectious treatment, immunosuppressors) was introduced in 65.5% of cases. However, three patients were not under specific treatment.

The mortality rate for HLH was 73.1%. Deaths occurred during hospitalization in 52% of the cases, and nine patients died after a remission period of approximately 5.8 months. The mean survival rate was 45 days. One case of HLH relapse was noted.

Analytical study

The study of the influencing outcome factors of HLH in our series is reported in Table 4. We noted that the male gender and the absence of etiological treatment more frequently correlated with mortality. On the other hand, the age of under 40 years and the absence of both hyperfibrinemia and underlying systemic disease were significantly correlated with patient survival. Furthermore, all patients with agranulocytosis (p: 0.221) or those without corticotherapy (p: 0.09) had higher mortality.

**Table 4:** Influencing outcome factors of HLH in our series HLH: hemophagocytic lymphohistiocytosis; OR: observed risk; CI: confidence interval

Factors	Patients died (n = 19)	Patients alive (n = 7)	P-value	OR (95% CI)
Age of <40 years	8	6	0.04	0.12 (0.01-1.21)
Male gender	17	1	0.000	51 (3.8-669.4)
Hemorrhagic syndrome	2	4	0.012	0.088 (0.011-0.71)
Normal or low fibrinemia	2	3	0.04	0.11 (0.011-1.14)
Renal function disorder	7	1	0.26	-
Myelodysplasia	9	3	0.78	-
Hematologic neoplasms	9	1	0.07	-
Systemic disease	1	4	0.003	0.04 (0.003-0.51)
Infections	4	2	0.68	-
Corticotherapy	13	7	0.09	-
Etoposide	5	2	0.90	-
Etiological treatment absence	11	1	0.04	8.2 (0.8-82.6)

## Discussion

HLH in adults is an uncommon condition recently labeled as a pathological entity [2]. In Sub-Saharan Africa, this pathology has been the subject of only a few case reports [3-5]. We conducted a three-year retrospective and cross-sectional study to collect 26 observations of adult HLH, undertaking the largest African patient series. In this study, we found that HLH generally affects adult male patients.

A variety of conditions including infection, malignancy, autoimmune disorders, and immunosuppression is associated with HLH in adults [10]. HLH occurred mainly in the presence of underlying hematological malignancies in our patients [2,11]. However, the association of acquired HLH with Hodgkin's disease, the main hematological malignancies found in our series, was rare [12]. HLH/autoimmune disease was diagnosed in five of our patients, with two cases of lupus. Regarding these predisposition fields, HLH in adults was mostly triggered by infection [13]. In our series, a documented infection was recorded in approximately half of the cases. They occurred individually or in a predisposition field: hematological malignancies and primary immunodeficiency.

HLH has a life-threatening clinical presentation that affects a wide range of organ systems [2]. An important presence of central neurological disorders was noted in this study. Typically, those neurological signs are more described in HLH in children [10]. However, it is difficult to determine the difference between signs related to HLH and those from the causal pathology. Nevertheless, the clinical assessment findings, as well as the cellularity of CSF, were normal for all those patients. The proportion of renal impairment in our series was high. Some cases of HLH were gathered in a nephrology unit, which could be a source of selection bias. The most specific histological sign noted in our study was collapsing focal segmental glomerulosclerosis. This abnormality was mainly reported in black African patients during acquired HLH [14,15].

Cytopenia was the main biological marker of HLH. Anemia, on the other hand, was profound and permanent in most cases. Thrombocytopenia was the most frequent precocious cytopenia of adult HLH [1,2]. However, this thrombocytopenia was recorded in only 65% of our patients. Paradoxical thrombocytosis was noted in three of our patients, with two cases of HLH/Hodgkin's disease and one with underlying lymphoma T. The association between HLH and thrombocytosis was rarely reported in the literature [3]. Thrombocytosis occurrence during HLH could be linked to a preexisting chronic inflammation or with underlying hematological malignancies such as Hodgkin's disease, in which the association with a thrombocytosis was reported [16]. Of the medullar hemophagocytosis, the myelogram highlighted dysmyelopoiesis in approximately half of our patients, which could be a cause or consequence of HLH. In fact, exceptional cases of myelodysplastic syndrome (MDS) complicated by HLH were reported [17]. Additionally, some cytological abnormalities mimicking MDS were described during HLH. They might be related to a cytokine rush that led to cellular damages resulting in morphological changes [18]. Although cytogenetic examinations were not performed in our patients, the hypothesis of an underlying MDS is unlikely with a mean age of 34 years and the disappearance of peripheral cytopenias with 42% having controlled HLH. The dysmyelopoiesis was recorded regardless of the context of vitamin B12 and/or folic acid deficiency. The dysmyelopoiesis, then, could be one more cytological argument for adult HLH diagnosis in our context.

HLH was also remarkable for the high mortality level in this study. This can be due to the diagnostic delay as well as the presence of underlying hematological malignancies. This analytical study helped identify some prognosis-associated factors of adult HLH. HLH in female patients aged younger than 40 years or with an underlying systemic disease was significantly associated with a good outcome. The absence of etiological treatment was the main poor outcome factor. On the other hand, the use of etoposide was not significantly associated with survival in our patients. Yet, it is presently the first-line treatment of adult HLH [11,19]. However, the efficacy of etoposide is dependent on the underlying etiology of HLH [11].

## Conclusions

We conducted the largest case series in a Sub-Saharan African population. Our study consisted of 26 observations, and our analysis revealed the particularities of adult HLH presentation in our population. The significant presence of neurological signs, myelodysplasia, and paradoxical thrombocytosis were important features of adult HLH in our study population. These features should be considered for the diagnostic assessment and management of HLH patients in tropical areas.
